# Genomic prediction in multi-environment trials in maize using statistical and machine learning methods

**DOI:** 10.1038/s41598-024-51792-3

**Published:** 2024-01-11

**Authors:** Cynthia Aparecida Valiati Barreto, Kaio Olimpio das Graças Dias, Ithalo Coelho de Sousa, Camila Ferreira Azevedo, Ana Carolina Campana Nascimento, Lauro José Moreira Guimarães, Claudia Teixeira Guimarães, Maria Marta Pastina, Moysés Nascimento

**Affiliations:** 1 Department of Statistics, Universidade Federal de Viçosa, Viçosa, Minas Gerais Brazil; 2 Department of General Biology, Universidade Federal de Viçosa, Viçosa, Minas Gerais Brazil; 3 Department of Mathematics and Statistics, Universidade Federal de Rondônia, Ji-Paraná, RO Brazil; 4 Embrapa Maize and Sorghum, Sete Lagoas, Minas Gerais Brazil

**Keywords:** Plant breeding, Genomics

## Abstract

In the context of multi-environment trials (MET), genomic prediction is proposed as a tool that allows the prediction of the phenotype of single cross hybrids that were not tested in field trials. This approach saves time and costs compared to traditional breeding methods. Thus, this study aimed to evaluate the genomic prediction of single cross maize hybrids not tested in MET, grain yield and female flowering time. We also aimed to propose an application of machine learning methodologies in MET in the prediction of hybrids and compare their performance with Genomic best linear unbiased prediction (GBLUP) with non-additive effects. Our results highlight that both methodologies are efficient and can be used in maize breeding programs to accurately predict the performance of hybrids in specific environments. The best methodology is case-dependent, specifically, to explore the potential of GBLUP, it is important to perform accurate modeling of the variance components to optimize the prediction of new hybrids. On the other hand, machine learning methodologies can capture non-additive effects without making any assumptions at the outset of the model. Overall, predicting the performance of new hybrids that were not evaluated in any field trials was more challenging than predicting hybrids in sparse test designs.

## Introduction

Maize (*Zea mays*) has emerged as an important crop for food, feed production, and various industrial applications, providing livelihoods for millions of people around the world^1,2^. However, its production is affected by several factors, with drought being one of the most common causes of agricultural shortages in rainfed systems^3^. This fact, combined with the high demand for this crop and the prospect of a worldwide growth of more than 2 billion people over the next 20 years^4^, makes it necessary to cultivate increasingly productive crops, as well as more adapted to climate change and also to different planting regions, such tropical conditions.

Cultivars can exhibit differentiated phenotypic responses between environments, and it is possible that a genotype may perform well in one environment but not in another^5–7^. To address this, breeders must submit the developed hybrids to multiple environment trials (MET). In MET, the main objectives are to study the interaction between genotypes and environments and to evaluate genotypic overall performance and stability^8^. However, MET phenotyping faces challenges such as the limited seeds availability, a high number of genotypes to be tested in the preliminary trials, and the associated costs, resulting in unbalanced experimental designs in different environments^9,10^. In sparse designs, where hybrids are not evaluated in all environments, accurately selecting superior hybrids for the next cycle can be difficult, as some hybrids may not be stable in many environments, and other genotypes that are discarded may outperform in untested environments.

To address these challenges, genomic prediction (GP) is proposed as a tool to predict the genetic value of individuals that were not evaluated in the field^11,12^. Several GP methods have been proposed, with Genomic Best Linear Unbiased Predictor (GBLUP) being one of the most commonly used methods. In the context of MET, predicting the genetic value of individuals not observed in specific environments has led to the development of several models, including interaction model of genotypes by environments, environmental covariates, and additive and dominance effects^9,13–16^.

Recently, machine learning methodologies have gained attention due to their ability to recognize complex interaction structures in data sets^17^. Machine learning algorithms approximate the mapping function linking input variables (e.g., phenotypic trait) to output variables from the training datasets without making a priori assumptions about data distribution or the genotype–phenotype relationship^18^. This flexibility allows these methods to capture more complex genetic architectures in prediction models.

Among the machine learning methodologies used in genomic prediction, decision trees and their refinements (such as bootstrap aggregation (bagging), random forest, and boosting) stand out, as they stratify the predictor space into many sub-regions^19,20^. These refinements aim to build more accurate prediction models; for example, bagging (Bag) reduces the variance observed in decision trees, random forest (RF) improves accuracy by avoiding high tree correlation^21^, and boosting (Boost) builds trees sequentially using information from previously built trees^22^.

Studies using simulated and real data have concluded that tree-based machine learning tools can serve as an alternative to traditional techniques for genomic prediction^23–26^. For instance, Sousa et al.^27^, evaluating genomic prediction for resistance to rust disease in *Coffea arabica*, observed that Bag, RF, and Boost showed superior predictive abilities to Generalized Bayesian Linear Regression. Westhues et al.^28^, using genomic and environmental variables, found that machine learning models can provide similar or slightly superior predictive abilities to GBLUP models for traits strongly influenced by environmental factors. Despite the potential of tree-based machine learning, there are still few studies that have evaluated MET data, and these methodologies could prove beneficial in such cases.

Therefore, our study aimed to evaluate and to compare the efficiency of statistical methodologies (GBLUP) and machine learning (Bag, RF, and Boost) for genomic prediction for single cross hybrid evaluated for drought tolerance traits in MET. We considered two different prediction scenarios, mimicking two situations that plant breeders may encounter: (i) predicting the performance of newly developed single hybrids for which there are no existing phenotypic records; (ii) predicting the performance of single cross hybrids in sparse design trials, where some hybrids were evaluated in some environments, but not in others.

## Material and methods

### Phenotypic data

The data are composed of 265 single cross hybrids from the maize breeding program of Embrapa Maize and Sorghum evaluated in eight combinations of trials/locations/years under irrigated trials (WW) and water stress (WS) conditions at two locations in Brazil (Janaúba—Minas Gerais and Teresina—Piauí) over two years (2010 and 2011). The hybrids were obtained from crosses between 188 inbred lines and two testers. The inbred lines belong to heterotic groups: dent (85 inbred lines), flint (86 inbred lines), and an additional group, referred to as group C (17 inbred lines), which is unrelated to the dent and flint origins. The two testers are inbred lines belonging to the flint (L3) and dent (L228-3) groups. Among the inbred lines, 120 were crossed with both testers, 52 were crossed with the L228-3 tester only, and 16 lines were crossed with the L3 tester only. Silva et al. (2020) evaluated the genetic diversity and heterotic groups in the same database. These authors showed the existence of subgroups within each heterotic group. Therefore, once these groups were not genetically well defined and the breeding program from Embrapa Maize was in the beginning, the effect of allelic substitution in both groups are assumed to be the same. More details on the experimental design and procedures can be found in Dias et al.^13,30^.

The experiment originally included 308 entries, but hybrids that were not present in all environments were also removed to evaluate the genomic prediction within each environment, resulting in a total of 265 hybrids for analysis. Each trial consisted of 308 maize single cross hybrids, randomly divided into six sets: sets 1–3 for crosses with L3 (61, 61, and 14 hybrids each), and sets 4–6 for crosses with L228-3 (80, 77, and 15 hybrids each). Four checks (commercial maize cultivars) were included in each set, and the experiment was designed in completely randomized blocks. Between trials, hybrids within each set remained the same, but hybrids and checks were randomly allocated into groups of plots within each set. This allocation varied between replicates of sets and between trials. The WS trials had three replications, except for the set containing 15 hybrids and the trials evaluated in 2010, which had two replications. All WW trials, except for the trial in 2011, had two replicates.

Two agronomic traits related to drought tolerance were analyzed: grain yield (GY) and female flowering time (FFT). GY was determined by weighing all grains in each plot, adjusted for 13% grain moisture, and converted to tons per hectare (t/ha), accounting for differences in plot sizes across trials. FFT was measured as the number of days from sowing until the stigmas appeared in 50% of the plants. A summary of means, standard deviations, and ranges of both evaluated traits are available in Table 1.

**Table 1:** Summary of means (Mean), standard deviations (SD), minimum (Min), and maximum (Max) for grain yield (GY) and female flowering time (FFT) obtained under irrigated (WW) and water stress (WS) conditions, evaluated in the years 2010 and 2011, at the locations of Janaúba (J) and Teresina (T).

	WS				WW			
	2010		2011		2010		2011	
	J	T	J	T	J	T	J	T
GY								
Mean	3.49	2.86	3.34	4.93	6.59	6.63	6.98	5.56
SD	1.34	1.65	1.22	1.63	1.63	1.70	1.68	1.92
Min	0.24	0.12	0.17	0.30	0.98	2.02	2.20	1.05
Max	8.36	9.57	6.82	9.42	11.55	12.49	10.86	10.24
FFT								
Mean	64.20	54.76	70.59	53.50	64.62	54.16	68.36	52.40
SD	2.61	5.30	3.25	2.54	3.07	2.77	2.19	2.10
Min	59.00	50.00	62.00	48.00	58.00	49.00	63.00	46.00
Max	73.00	69.00	88.00	64.00	74.00	64.00	75.00	59.00

To conduct the analyses, hybrids considered as outliers were removed (i.e., hybrids that presented phenotypic values greater than 1.5 × interquartile range above the third quartile or below the first quartile) for the GY and FFT traits. The variations in predictive abilities among hybrids of T2, T1, and T0 are widely recognized^31^. However, the primary aim of our study was to compare different prediction methodologies in MET assays. In this study, there were 240 T2 hybrids and 68 T1 hybrids, with T2 hybrids had both parents evaluated in different hybrid combinations, while hybrids being single-cross hybrids sharing one parent with the tested hybrids. Given the realistic nature of our scenario, we have a limited and imbalanced distribution of these hybrid groups, making a fair comparison challenging. Consequently, we opted to construct a training set comprising T2 and T0 hybrids.

### Statistical analysis of phenotypic data

To correct the phenotypic values for experimental design effects, each trial (WW and WS) and environment were analyzed independently to obtain the Best Linear Unbiased Estimator (eBLUEs) for each hybrid, for the two traits evaluated. The estimates were obtained based on the following model:

1 $${\varvec{y}} = 1\mu + \user2{ X}_{1} {\varvec{r}} + {\varvec{X}}_{2} {\varvec{s}} + {\varvec{X}}_{3} {\varvec{h}} + {\varvec{e}}$$

where $\user2{y }\left( {n \times 1} \right)$ is the phenotype vector for $f$ replicates, $t$ sets of $p$ hybrids, and $n$ is the number of observations; $\mu$ is the mean; $\user2{r }\left( {f \times 1} \right)$ is the fixed effect vector of the replicates; $\user2{s }\left( {t \times 1} \right)$ is the fixed effect vector of the sets; ${\varvec{h}}$ $\left( {p \times 1} \right)$ is the fixed effect vector of the hybrids; and $\user2{e }\left( {k \times 1} \right)$ is the residue vector, with $\user2{ e} \sim ,MVN\left( {0,{\varvec{I}}\sigma_{e}^{2} } \right)$, where ${\varvec{I}}$ is an identity matrix of corresponding order, and $\sigma_{e}^{2}$ the residual variance. ${\varvec{X}}_{{1\user2{ }}} \left( {k \times f} \right)$**,** ${\varvec{X}}_{{2\user2{ }}} \left( {k \times t} \right)$ e ${\varvec{X}}_{{3\user2{ }}} \left( {k \times p} \right)$ represents incidence matrices for their respective effects. The eBLUES of each environment were used in further analyses.

### Genotypic data

A total of 57,294 Single Nucleotide Polymorphisms (SNPs) markers were obtained from 188 inbred lines, and two testers used as parents of the 265 single cross hybrids. The genotyping by sequencing (GBS) strategyare detailed in Dias et al.^13^. For the quality control, SNPs were discarded if: the minor allele frequency was smaller than 5%, more than 20% of missing genotypes were found, and/or there were more than 5% of heterozygous genotypes. After filtering, missing data were imputed using NPUTE. Then, for each SNP, the genotypes of the hybrids were inferred based on the genotype of their parents (inbred line and tester). The number of SNPs per chromosome ranged from 3121 (chromosome 10) to 7705 (chromosome 1), totalizing 47,127 markers.

### Genomic relationship matrix

The additive and dominance genomic relationship matrices were constructed^32^ based on information from the SNPs using the package AGHmatrix^33^, following VanRaden^34^ and Vitezica et al., respectively.

### Genomic prediction

Genomic predictions were performed using the Genomic Best Line Unbiased Prediction (GBLUP) method using the package AsReml v. 4^36^. Two groups were considered: the first group comprised four environments under WW conditions, and the second included four environments under WS conditions. The linear model is described below:

2 $$\overline{\user2{y}} = \mu 1 + \user2{ Xb} + {\varvec{Z}}_{1} {\varvec{u}}_{{\varvec{a}}} + {\varvec{Z}}_{2} {\varvec{u}}_{{\varvec{d}}} + {\varvec{e}}$$

where $\user2{\overline{y} }\left( {pq \times 1} \right)$ is the vector of eBLUES previously estimated for each environment with $p$ hybrids and $q$ environments;$\mu$ is the mean; $\user2{b }\left( {q \times 1} \right)$ is the vector of environmental effects (fixed); ${\varvec{u}}_{{\user2{a }}} \left( {pq \times 1} \right)$ is the vector of individual additive genetic values nested within environments (random), with ${\varvec{u}}_{{\varvec{a}}} \sim MVN\left( {0,\left[ {{\varvec{I}}_{{\varvec{q}}} \sigma_{{u_{a} }}^{2} + \rho_{a} \left( {{\varvec{J}}_{{\varvec{q}}} - {\varvec{I}}_{{\varvec{q}}} } \right)} \right] \otimes {\varvec{A}}} \right)$, where ${\varvec{A}}$ is the genomic relationship matrix between individuals for additive effects, $\rho_{a}$ is the additive genetic correlation coefficient between environments, ${\varvec{I}}_{{\varvec{q}}} \user2{ }\left( {q \times q} \right)$ is an identity matrix, ${\varvec{J}}_{{\varvec{q}}} \user2{ }\left( {q \times q} \right)$ is a matrix of ones, and $\otimes$ denotes the Kronecker product; ${\varvec{u}}_{{\varvec{d}}}$ $\left( {pq \times 1} \right)$ is the vector of individual dominance genetic values nested within environments (random), with ${\varvec{u}}_{{\varvec{d}}} \sim MVN\left( {0,\left[ {{\varvec{I}}_{{\varvec{q}}} \sigma_{{u_{d} }}^{2} + \rho_{d} \left( {{\varvec{J}}_{{\varvec{q}}} - {\varvec{I}}_{{\varvec{q}}} } \right)} \right] \otimes {\varvec{D}}} \right)$, where ${\varvec{D}}$ is the genomic relationship matrix between individuals for dominance effects, $\rho_{{\varvec{d}}}$ is the dominance correlation coefficient between environments; ${\varvec{e}}$ $\left( {pq \times 1} \right)$ is the random residuals vector with ${\varvec{e}}\sim MVN\left( {0,{\varvec{I}}\sigma_{e}^{2} } \right)$. The capital letters $\user2{X }\left( {pq \times q} \right),\user2{ Z}_{1} \left( {pq \times pq} \right)$ and ${\varvec{Z}}_{2} \user2{ }\left( {pq \times pq} \right)$ represent the incidence matrices for their respective effects, $1\user2{ }\left( {pq \times 1} \right)$ is a vector of ones. The (co)variance components were obtained using the residual maximum likelihood method (REML)^37^.

Two alternative models were also used. The first for genomic prediction retained only additive effects by removing ${\varvec{u}}_{{\varvec{d}}}$ from Eq. (2). The second model was used to estimate the genetic parameters within each environment separately.

The significance of random effects was tested using the Likelihood Ratio Test (LRT)^38^, given by:

3 $$LRT = 2*\left( {LogL_{c} - LogL_{r} } \right)$$

where $LogL_{c}$ is the logarithm of the likelihood function of the complete model (with all effects included), and $LogL_{r}$ is the logarithm of the restricted likelihood function of the reduced model (without the effect under test). Effect significance was tested by LRT using the chi-square (X^2^) probability density function with a degree of freedom and significance level of 5%^39^.

The narrow-sense heritability (${ }h^{2}$), the proportion of variance explained by dominance effects ($d^{2}$), and the broad-sense heritability $\left( {H^{2} } \right)$ for each trait were estimated following Falconer and Mackay 1996^35^.

### Machine learning

Similar to the previous topic, the trials were divided between WW and WS conditions, and the potential of regression trees (RT) was explored using the following three algorithms: bagging, random forest, and boosting^22^. Bagging (Bag) is a methodology that aims to reduce the RT variance^22^. In other words, it consists of obtaining D samples with available sampling replacement, thus obtaining D models $\hat{f}^{1} \left( x \right), \hat{f}^{2} \left( x \right), \ldots , \hat{f}^{D} \left( x \right)$, and finally use the generated models to obtain an average, given by:

4 $$\hat{f}_{medio} \left( x \right) = \frac{1}{D}\mathop \sum \limits_{d = 1}^{D} \hat{f}^{d} \left( x \right)$$

This decreases the variability obtained in the decision trees. The number of trees used in Bag is not a parameter that will result in overfitting of the model. In practice, a number of trees is used until the error has stabilized^22^. The number of trees sampled for Bag was set at 500 trees.

Random forest (RF) was proposed by HO^40^ and it is an improvement of Bag to avoid the high correlation of the trees and to improve the accuracy in the selection of individuals. RF changes only the number of predictor variables used in each split. That is, each time a split in a tree is considered, a random sample of $m$ variables is chosen as candidates from the complete set of $p$ variables. Hastie et al.^21^ suggest that the number of predictor variables used in each partition is equal to $m = \frac{p}{3}$ for regression trees. The number of trees for the RF was set at 500.

Boosting uses RT by adjusting the residual of an initial model. The residual is updated with each tree that grows sequentially from the previous tree's residual, and the response variable involves a combination of a large number of trees, such that:

5 $$\hat{f}\left( x \right) = \mathop \sum \limits_{b = 1}^{B} {\uplambda } \hat{f}^{b} \left( x \right)$$

The function $\hat{f}\left( . \right)$ refers to the final tree combined with sequentially adjusted trees, and λ is the shrinkage parameter that controls the learning rate of the method. Furthermore, this method needs to be adjusted with several splits in each of the trees. This parameter controls the complexity of the Boost and is known as the depth. For Boosting, the number of trees sampled was 250, with a learning rate of 0.1 and a depth of 3.

To perform hybrid prediction for each environment based on MET dataset, we propose the incorporation of location and year information in which the experiments were carried out as factors in the data input file together with SNPs markers as predictors in machine learning methodologies. As a response variable, the eBLUEs previously estimated by Eq. (1) were used.

For the construction of the bagging and random forest models, the *randomForest* function from the package randomForest^41^ was used. Finally, the package's gbm function *gbm*^42^ was used for boosting. All analyzes were implemented in the software R^43^.

### Model validation

Genomic predictions were carried out following Burgueño et al.^16^, considering two different prediction problems, CV1 and CV2, which simulate two possible scenarios a breeder can face. In CV1, the ability of the algorithms to predict the performance of hybrids that have not yet been evaluated in any field trial was evaluated. Thus, predictions derived from the CV1 scenario are entirely based on phenotypic and genotypic records from other related hybrids. In CV2, the ability of the algorithms to predict the performance of hybrids using data collected in other environments was evaluated. It simulates the prediction problem found in incomplete MET trials. Here, information from related individuals is used, and the prediction can benefit from genetic relationships between hybrids and correlations between environments. Within the CV2 scenario, two different situations of data imbalance were evaluated. In the first, called CV2 (50%), the tested hybrids were not present in half of the environments, while in the second, called CV2 (25%), the tested hybrids were not present in only 25% of the environments. Table 2 provides a hypothetical representation of this CV1, CV2 (50%), and CV2 (25%) validation scheme.

**Table 2:** Representation of the three scenarios (CV1, CV2-50% and CV2-25%) for four hybrids (Hybrid 1–4) and four environments (J10, J11, T10, T11).

	J10	J11	T10	T11
CV1				
Hybrid 1	H_11_	H_12_	H_13_	H_14_
Hybrid 2	NA	NA	NA	NA
Hybrid 3	H_31_	H_32_	H_33_	H_34_
Hybrid 4	H_41_	H_42_	H_43_	H_44_
CV2 (50%)				
Hybrid 1	H_11_	H_12_	H_13_	H_14_
Hybrid 2	NA	H_22_	H_23_	NA
Hybrid 3	H_31_	H_32_	H_33_	H_34_
Hybrid 4	H_41_	NA	NA	H_44_
CV2 (25%)				
Hybrid 1	H_11_	H_12_	H_13_	NA
Hybrid 2	NA	H_22_	H_23_	H_24_
Hybrid 3	H_31_	H_32_	NA	H_34_
Hybrid 4	H_41_	NA	H_43_	H_44_

Hybrids with phenotypes not observed in the scenario are indicated with NA (not evaluated); Hybrids with observed phenotypes are named as H_ij_ for *i,j* = 1, 2, 3 e 4.

To separate the training and validation sets, the k-folds procedure was used, considering $k = 5$. The set of 265 hybrids was divided into five groups, with 80% of the hybrids considered as the training population, and the remaining 20% hybrids considered as the validation population. The hybrids were separated into sets proportionally containing all the crosses performed (*Dent* × *Dent*, *Dent* × *Flint*, *Flint* × *Flint*, C × *Dent*, C × *Flint*). The cross-validation process was performed separately for each trait, condition (WS or WW) and scenario (CV1, CV2-50% and CV2-25%) and was repeated five times to assess the predictive ability of the analyses.

The predictive ability within each environment for the conditions (WS and WW) was estimated by the Pearson correlation coefficient^44^ between the corrected phenotypic values (eBLUES) of Eq. (1) for each environment and the GEBVs predicted by each fitted method.

### Ethics statement

The authors confirm that all methods were carried out by relevant guidelines in the method section. The authors also confirm that the handling of the plant materials used in the study complies with relevant institutional, national, and international guidelines and legislation.

### Statement of handling of plants

The authors confirm that the appropriate permissions and/or licenses for collection of plant or seed specimens are taken**.**

## Results

### Variance components and estimation of genetic parameters

Estimates of variance components and genetic parameters for GY and FFT under WW and WS conditions, obtained for the joint analysis with the four environments and analyses within each environment, are shown in Table 3 . For the joint analysis, the heritability estimates for GY and FFT were slightly different from those obtained by Dias et al.^13^ using the same material, since here, a different statistical model was used to estimate the genetic parameters, and hybrids that were not present in all environments were removed.

**Table 3:** Estimates of variance components and genetic parameters for grain yield (GY) and female flowering time (FFT) were obtained considering the joint analysis for the four evaluated environments and analyses within each environment, for the irrigated (WW) and water stress (WS) conditions.

	WS	WW	WS				WW			
			2010		2011		2010		2011	
			Janaúba	Teresina	Janaúba	Teresina	Janaúba	Teresina	Janaúba	Teresina
*GY*										
$\hat{\sigma }_{{u_{a} }}^{2}$	0.60*	1.00*	0.64*	0.88*	0.35*	0.50*	1.47*	1.15*	1.59*	0.96*
$\hat{\sigma }_{{u_{d} }}^{2}$	0.30*	0.45*	0.39*	0.11^ ns^	0.34*	0.71*	0.56*	0.61*	1.42*	0.45^ ns^
$\hat{\sigma }_{e}^{2}$	0.81	1.98	0.66	1.61	0.33	0.44	0.53	1.02	1.83	3.30
$\rho_{a}$	0.35	0.24	–	–	–	–	–	–	–	–
$\rho_{d}$	0.83	0.85	–	–	–	–	–	–	–	–
*h*^*2*^	0.35	0.29	0.38	0.34	0.34	0.30	0.57	0.41	0.33	0.20
*d*^*2*^	0.17	0.13	0.23	0.04	0.33	0.43	0.22	0.22	0.29	0.10
*H*^*2*^	0.53	0.42	0.61	0.38	0.68	0.73	0.79	0.63	0.62	0.30
FFT										
$\hat{\sigma }_{{u_{a} }}^{2}$	4.66*	3.13*	6.86*	1.54*	6.85*	2.04*	4.24*	2.93*	3.42*	2.38*
$\hat{\sigma }_{{u_{d} }}^{2}$	1.16*	0.82*	0.75*	0.12^ ns^	1.88*	1.20*	0.83*	1.15*	0.45^ ns^	1.03*
$\hat{\sigma }_{e}^{2}$	4.54	1,64	0.97	14.73	1.73	1.40	0.69	1.88	2.70	1.04
$\rho_{a}$	0.64	0.82	–	–	–	–	–	–	–	–
$\rho_{d}$	0.43	0.37	–	–	–	–	–	–	–	–
*h*^*2*^	0.45	0.56	0.80	0.09	0.65	0.44	0.74	0.49	0.52	0.54
*d*^*2*^	0.11	0.15	0.09	0.01	0.18	0.26	0.14	0.19	0.07	0.23
*H*^*2*^	0.56	0.71	0.89	0.10	0.83	0.70	0.88	0.68	0.59	0.77

$\hat{\sigma }_{{u_{a} }}^{2}$: additive genetic variance; $\hat{\sigma }_{{u_{d} }}^{2}$: dominance genetic variance; $\hat{\sigma }_{e}^{2}$: residual variance; $\rho_{a:}$ additive genetic correlation coefficient between environments, $\rho_{d} :$ dominance genetic correlation coefficient between environments; *h*^*2*^*:* narrow sense heritability; *d*^*2*^: proportion of the variance explained by the dominance effect and* H*^*2*^*:* broad sense heritability*.* ^ns^ and *, not significant and significant at 5% probability of error.

The additive variance found for GY and FFT was greater than the variance due to dominance effects, in both WS and WW conditions. For GY, the variances due to dominance effects represented about 33.3% and 31.0% of the genetic variance in WS and WW conditions, respectively. Lower broad-sense heritability was observed for this trait in WW (0.42) when compared to the WS condition (0.53). As for FFT, the variances due to dominance effects represented about 19.9% and 20.8% of the genetic variance in WS and WW conditions, respectively, and the broad-sense heritabilities for FFT were greater in WW conditions (0.71) than in conditions WS (0.56).

For GY, the narrow-sense heritabilities within environments ranged from 0.30 (T11) to 0.38 (J10) under WS conditions and from 0.20 (T11) to 0.57 (J10) under WW conditions. The proportion of genetic variance explained by dominance deviations ranged from 0.04 (T10) to 0.43 (T11) under WS conditions and from 0.10 (T11) to 0.29 (J11) under WW conditions. The broad-sense heritabilities were lower for the experimental tests that had a lower number of repetitions (2010 under WS conditions, and 2011 under WW conditions).

For FFT, the narrow-sense heritabilities ranged from 0.09 (T10) to 0.80 (J10) under WS conditions and from 0.49 (T10) to 0.74 (J10) under WW conditions. The proportion of genetic variance explained by dominance deviations ranged from 0.01 (T10) to 0.26 (T11) under WS conditions and from 0.07 (J11) to 0.23 (T11) under WW conditions. The broad-sense heritabilities were higher for J10 (0.89 and 0.88) under WS and WW conditions.

The Eq. (2) is an implicit model to perform MET analyses^45^ and provide genetic correlations for additive and dominance effects across environments. This model, reflects on the levels of genotypes-by-environment interaction. Particularly, for GY, the environmental correlations were 0.35 and 0.24 for WS and WW conditions, respectively, indicating an inconsistent ranking of hybrids across environments. As for FFT, the lowest correlation was observed for dominance effects.

### Efficiency of prediction methodologies in multi-environment trials

**Figure 1:**
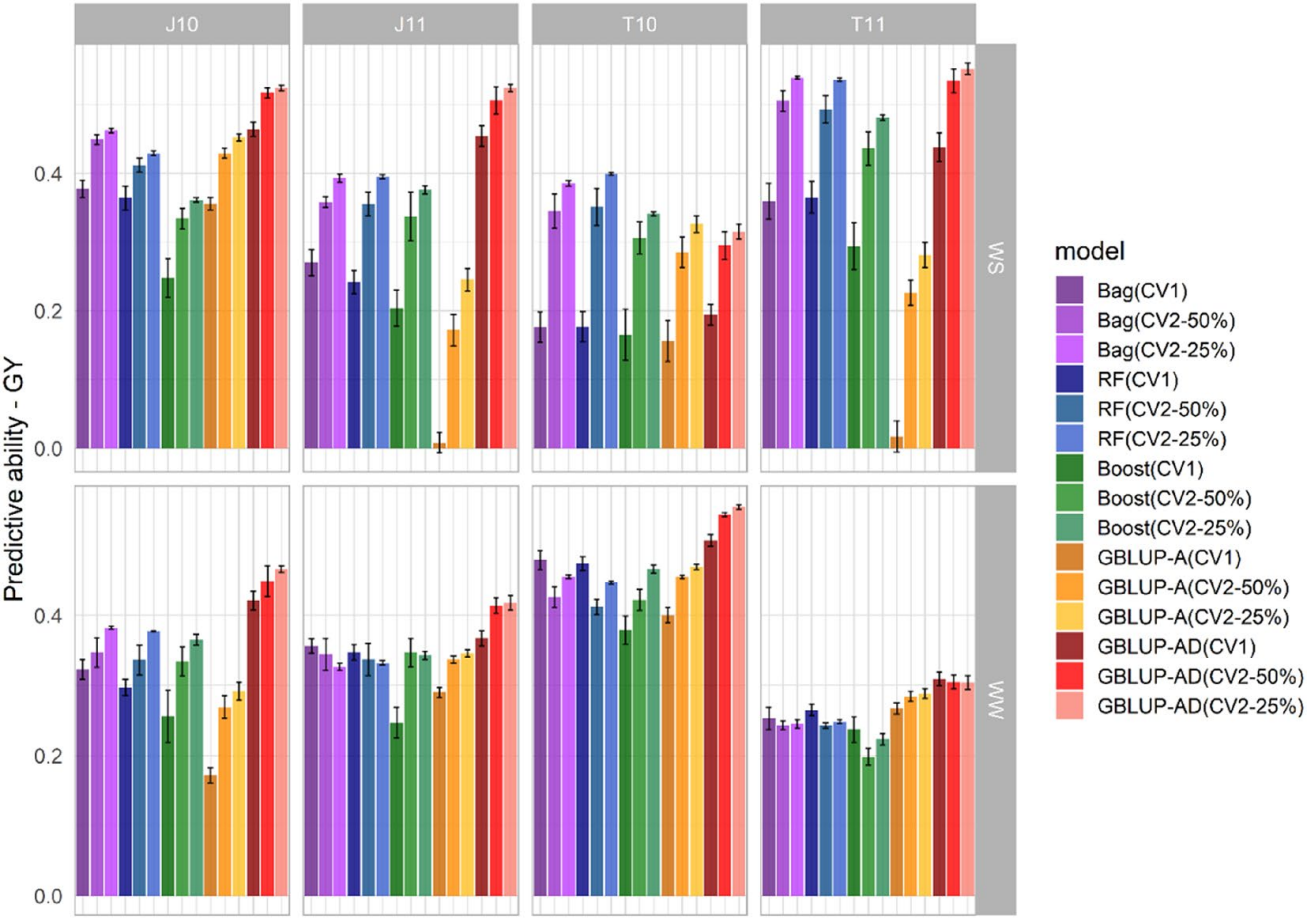
Mean predictive abilities and their respective standard errors for grain yield (GY), evaluated in the environments of Janaúba/2010 (J10), Janaúba/2011 (J11), Teresina/2010 (T10) and Teresina/2011 (T11)), under the CV1, CV2 (50%) and CV2 (25%) scenarios, considering irrigated (WW) and water stress (WS) conditions. The evaluated methodologies include bagging (Bag), random forest (RF), boosting (Boost), GBLUP additive model (GBLUP-A), and GBLUP additive-dominant model (GBLUP-AD).

**Figure 2:**
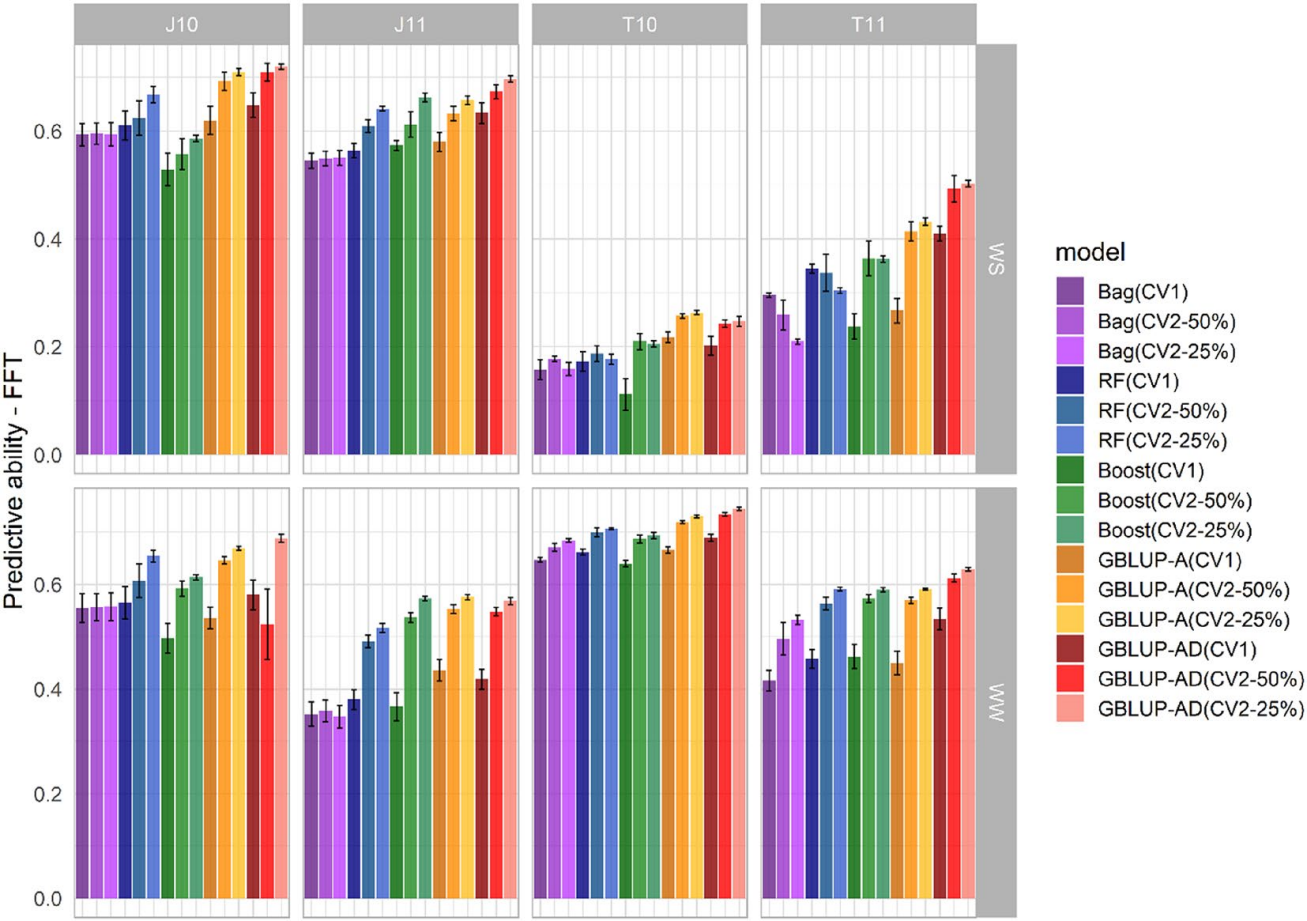
Mean predictive abilities and their respective standard errors for female flowering time (FFT), evaluated in the environments of Janaúba/2010 (J10), Janaúba/2011 (J11), Teresina/2010 (T10) and Teresina/2011 (T11), under CV1, CV2 (50%) and CV2 (25%) scenarios, considering irrigated (WW) and water stress (WS) conditions. The evaluated methodologies include bagging (Bag), random forest (RF), boosting (Boost), GBLUP additive model (GBLUP-A), and GBLUP additive-dominant model (GBLUP-AD).

Figures 1 and 2 show the predictive abilities observed in the three scenarios (CV1, CV2-50%, and CV2-25%) for each of the five compared methods: GBLUP additive model (GBLUP-A), GBLUP additive-dominant model (GBLUP-AD), bagging (Bag), random forest (RF) and boosting (Boost). The numerical results of these figures are presented in Supplementary Tables 1, 2, and 3.

For the GBLUP models, the predictive abilities were lower for the CV1 scenario, where the predictions were based only on phenotypic and genotypic records of other related hybrids. However, the predictive ability increased when the predicted hybrid was present in some environments, being intermediate in CV2-50% and higher in CV2-25%. A more notable improvement in predictive abilities was observed when transitioning from CV1 to CV2-50%. Specifically, for GBLUP-A, the average increase was about 107%, 19%, 18%, and 19% for GY-WS, GY-WW, FFT-WS, and FFT-WW, respectively. As for GBLUP-AD, the average increase was 19%, 7%, 12%, and 15% for GY-WS, GY-WW, FFT-WS, and FFT-WW, respectively (Table Sup. 4). For GY, considering only CV1 scenario, the average predictive abilities were higher in WW conditions. For FFT, mean predictive abilities for WW conditions were higher than for WS for all scenarios (Table Sup. 4).

The environments with the highest broad-sense heritabilities also exhibited the highest average predictive abilities across all evaluated methodologies (Table 3, Figs. 1 and 2). The GBLUP-AD model demonstrated superior predictive abilities for the GY and FFT traits in almost all environments and scenarios (CV1, CV2-50%, and CV2-25%). Conversely, the GBLUP-A performed equally or better than the GBLUP-AD model for FFT in environments where the dominance effect was not significant (T10-WS and J11-WW). However, for GY, in environments where the proportion of dominance genetic variance was close to or greater than the additive variance (J11 and T11 under WS), GBLUP-A exhibited lower predictive ability.

Unlike GBLUP, machine learning methodologies did not show a consistent pattern of increased predictive ability with the presence of phenotypic records of the hybrid to be predicted in correlated environments. Overall, an increase in predictive abilities for GY was observed when the scenario changed from CV1 to CV2, under WS conditions, which was not observed in many environments under WW conditions. For example, for RF, the average predictive abilities in the environments increased by approximately 51% for GY in WS conditions, while a decrease of about 10% was observed when the prediction changed from CV1 to CV2-50% for the same trait in WW conditions (Table Sup. 4). As for FFT, Bag drew the most attention, presenting a large standard error in predictive abilities with statistically similar results for CV1, CV2-50% and CV2-25% in WS and WW conditions, in almost all environments.

In environments where dominance effects accounted for a large portion of the genetic variance, the Bag, RF and Boost methodologies showed intermediate results between the two GBLUP models. Among the evaluated machine learning methodologies, Bag and RF produced very similar predictions and Boost presented the lowest predictive ability for GY. As for FFT, machine learning did not show improvement in predictive abilities when compared to GBLUP.

## Discussion

We employed both statistical and machine learning methods to evaluate the efficiency of genomic predictions for GY and FFT. These models allow us to leverage information about relationships between hybrids and correlated environments. Three scenarios, CV1, CV2-50% and CV2-25%, were used, each characterized by different degrees of data imbalance. Predicting the performance of new hybrids that have not been evaluated in the field (CV1) is more challenging compared to predicting hybrids that have been evaluated in an unbalanced manner across correlated environments (CV2-50% and CV2-25%)^15,16^.

Among the evaluated methodologies, GBLUP stands out as it allows to estimate relationships between individuals based on molecular markers for additive and dominant effects^32,34^. This methodology also allows to incorporate variance–covariance structures to handle correlated environments and unbalanced data^10,14^. As expected, GBLUP showed the highest predictive abilities in the CV2 validation schemes (CV2-50% and CV2-25%) as the predictions benefited from phenotypic records of the same hybrids in other correlated environments. Similar results have been reported in previous studies using wheat inbred lines and maize single cross hybrids^13–16^.

GBLUP-AD demonstrated greater efficiency to predict hybrids for traits and environments in which the effects due to dominance deviations were significant. As a substantial part of the genetic variance in maize hybrids for GY is attributed to dominance effects^46^, incorporating theses effects in the model significantly improved the prediction of this trait. However, when the dominance effect accounts for a small portion of the genetic variance (as observed for FFT), additive and additive-dominant models tend to show minor improvements in the predictions of the estimated total genetic effects^13,47^. This emphasizes the importance of understand the genetic bases of hybrids which can be decomposed into general combining ability (GCA) and specific combining ability (SCA). For GCA, the additive variance is the major component of the variance^48^ and, SCA is largely dependent on genes with dominance or epistatic effects^49^. In this context, gaining a comprehension of the relative magnitudes of GCA and SCA variations is instrumental in guiding the formulation of optimal strategies for hybrid breeding programs, as highlighted in previous research^50^.

One possible reason why tree-based learning models did not benefit from the presence of phenotyped hybrids in correlated environments is related to their shared concept of recursive division^51^. These models aim to find decision rules that naturally partition the prediction space to provide an informative and robust hierarchical model^21,52^. In this context, it is conceivable that the location/year variables played a crucial role in most of the tree construction scenarios, leading to the split of the same hybrids at the beginning of branching and making it difficult for them to be grouped at the last nodes, which are responsible for the predictive response.

Among the evaluated methodologies, Bag showed, in most environments, the same prediction pattern for both scenarios where the predicted hybrid was absent in all environments and where it was absent only in some environments. Bag is a variation of the decision tree that uses resampling of the original data in subsamples (bootstrap) according to the determined number of trees. This process may lead to high correlation between the generated trees, resulting in the same variable consistently being the most important one^21^. As consequence, the same hybrids in different environments may end up at very distant nodes in the construction of the tree. The RF further reduces the dependence between the decision trees by randomly selecting part of the original data and variables to build the trees^22,51^. This allows different variables to have a chance to be at the top in their construction. On the other hand, boost sequentially combines different predictors, fitting a new tree to the residuals of the previous model using a specified loss function (e.g. mean squared error for regression)^53^. It incorporates automatic indirect selection of markers and is generally recommended for regression analysis^17,20^. It is important to note that these variations of decision trees mentioned above are typically used when obtaining accurate predictions is more important than the biological interpretation of the model itself^52^.

One of the advantages of using machine learning methodologies is that they do not require the specification of the trait inheritance, having as an initial hypothesis the possibility of capturing non-additive effects in the genome, which are often not specified in traditional statistical methods^25^. Possibly tree-based machine learning methodologies managed to capture part of the dominance effect, presenting better results than GBLUP-A in environments where dominance represented a large part of the genetic variance. These methodologies are competitive with statistical models and tend to outperform them when applied to large data sets. However, when applied to small training sets, machine learning is probably not able to capture all non-additive information and linear models may perform better^54^.

Tree-based machine learning methodologies (Bag, RF, and Boost) are considered promising for genomic prediction, especially for traits controlled by a few quantitative trait locus (QTL), capturing non-additive effects in their models^25^. Among the machine learning methodologies evaluated in this study, Boost is considered the most sensitive methodology for the variation of traits and environments^25,26,55^. In these studies, carried out with simulated data, the lower the heritability and the greater the number of QTL that control the trait, the lower the Boost prediction efficiency. This fact may be related to the lower values of predictive ability for GY when compared to other methodologies, since this is a quantitative trait with low heritability, greatly influenced by the environment^56,57^.

A possible explanation for the lower values of predictive ability of machine learning methodologies when compared to GBLUP in FFT prediction is that, even though it is a complex trait^58,59^, additive genetic variance contributed with a large proportion of this trait. If the relationship between trait and response is close to a linear model, then a linear approach will probably work well and be superior to a method like a regression tree that does not explore this linear structure^22,60^.

In the context of a hybrid breeding program, identifying high-performance genotypes is essential^61^. However, extensive field trials are needed to identify the best hybrids in the target environment. These trials require resources in terms of people, equipment, and area to carry out the phenotyping of the plants. Furthermore, most crosses are discarded after evaluation due to poor overall performance^62^. Associated with this fact is the trend of stagnating or rising costs of field evaluations, unlike genotyping which tends to decrease^63^. In this sense, the use of genomic information in multiple-environment trials is an alternative to traditional field trials, as it allows to reduce the phenotyping costs and to increase the number of hybrid combinations evaluated when performing the prediction of the genetic value of individuals that were not evaluated in the field^11,64^.

Based on the results of this study, a practical application of cost reduction and the efficiency of genomic selection in a breeding program can be demonstraded^10^. Assuming the cost of an experimental maize trial is $17.00 per plot^65^ and the cost of sequencing genotyping (GBS) standard is $25.38 per parental line^66^, the total budget needed for a breeding program would be $108,120.00 for phenotyping the 265 single cross hybrids in eight experimental trials using three replications, and $5,076.00 for genotyping the 188 parental lines and the two testers. Using the GBLUP-AD model, it was shown that the predictive abilities for the untested single hybrids averaged greater than 0.40 for GY under WW conditions, with an imbalance of 25% of hybrids randomly missing in each environment. Consequently, the cost reduction for the breeding program would be approximately 25% or $27,030.00 compared to phenotyping alone, which would cover the costs of genotyping the lines ($5,076.00).

The models proposed in this study can be applied to other crops that use hybrids to explore heterosis, and they can be expanded to include environmental variables to predict non-evaluated environments. In addition, this study demonstrates the application of machine learning methodologies in tests of multiple environments for predicting of hybrids, comparing their performance with GBLUP with non-additive effects, thus highlighting the potential of both methodologies.

## Conclusion

Genomic prediction for untested maize single cross hybrids using both statistical and machine learning approaches were applied in MET assays. The results demonstrate that both methodologies are efficient and can be valuable tools in maize breeding programs to accurately predict the performance of hybrids in specific environments. The choice of the best methodology depends on the specific case. To optimize the predictive ability of GBLUP, it is crucial to accurately model the variance components. On the other hand, machine learning methodologies have the advantage of capturing non-additive effects without making any assumptions at the outset of the model. We found that predicting the performance of new hybrids that were not evaluated in any field trials was more challenging than predicting hybrids in unbalanced trials. However, regardless of the methodology used, environments with the lowest heritability showed the lowest predictive abilities, underscoring the importance of conducting well-designed and properly replicated experiments.

### Supplementary Information

Supplementary Tables.

## Data Availability

Data available in the Dryad Digital Repository: https://doi.org/10.5061/dryad.ps22r.

## References

[1] Hossain, F. *et al.* Molecular breeding for increasing nutrition quality in maize: recent progress. In *Molecular Breeding in Wheat, Maize and Sorghum: Strategies for Improving abiotic Stress Tolerance and Yield* 360–379 (CABI, 2021). 10.1079/9781789245431.0021.

[2] Hossain, F. *et al.* Maize Breeding. in *Fundamentals of Field Crop Breeding* 221–258 (Springer Nature Singapore, 2022). 10.1007/978-981-16-9257-4_4.

[3] Lobell DB (2014). Greater sensitivity to drought accompanies maize yield increase in the U.S. midwest. Science.

[4] ONU. World Population Prospects 2022. https://population.un.org/wpp/Graphs/Probabilistic/POP/TOT/900 (2022).

[5] Cruz, C. D., Regazzi, A. J. & Carneiro, P. C. S. Modelos biométricos aplicados ao melhoramento. *UFV, Viçosa* (2012).

[6] Malosetti M, Ribaut J-M, van Eeuwijk FA (2013). The statistical analysis of multi-environment data: Modeling genotype-by-environment interaction and its genetic basis. Front. Physiol..

[7] Crossa, J. Statistical Analyses of Multilocation Trials. in 55–85 (1990). 10.1016/S0065-2113(08)60818-4.

[8] Burgueño J, Crossa J, Cotes JM, Vicente FS, Das B (2011). Prediction assessment of linear mixed models for multienvironment trials. Crop Sci..

[9] Jarquin D (2020). Genomic prediction enhanced sparse testing for multi-environment trials. G3 Genes Genomes Genetics.

[10] Krause MD (2020). Boosting predictive ability of tropical maize hybrids via genotype-by-environment interaction under multivariate GBLUP models. Crop Sci..

[11] Bernardo R (1994). Prediction of maize single-cross performance using RFLPs and information from related hybrids. Crop Sci..

[12] Meuwissen THE, Hayes BJ, Goddard M (2001). Prediction of total genetic value using genome-wide dense marker maps. Genetics.

[13] Dias KODG (2018). Improving accuracies of genomic predictions for drought tolerance in maize by joint modeling of additive and dominance effects in multi-environment trials. Heredity.

[14] Jarquin, D. *et al.* Increasing genomic‐enabled prediction accuracy by modeling genotype × environment interactions in Kansas wheat. *Plant Genome* **10**, (2017). 10.3835/plantgenome2016.12.0130 28724062

[15] Jarquin D (2014). A reaction norm model for genomic selection using high-dimensional genomic and environmental data. Theor. Appl. Genet..

[16] Burgueño J, Campos G, Weigel K, Crossa J (2012). Genomic prediction of breeding values when modeling genotype × environment interaction using pedigree and dense molecular markers. Crop Sci..

[17] González-Recio O, Rosa GJM, Gianola D (2014). Machine learning methods and predictive ability metrics for genome-wide prediction of complex traits. Livest. Sci..

[18] Zhou Z-H (2021). Machine Learning.

[19] Jannink J-LJ-L, Lorenz AJ, Iwata H (2010). Genomic selection in plant breeding: from theory to practice. Brief. Funct. Genomics.

[20] Ogutu JO, Piepho H-P, Schulz-Streeck T (2011). A comparison of random forests, boosting and support vector machines for genomic selection. BMC Proc..

[21] Hastie T, Tibshirani R, Friedman J, Cruz CD, Nascimento M (2009). The Elements of Statistical Learning: Data Mining, Inference, and Prediction.

[22] Gareth J, Daniela W, Trevor H, Robert T (2013). An Introduction to Statistical Learning: with Applications in R.

[23] Sarkar RK, Rao AR, Meher PK, Nepolean T, Mohapatra T (2015). Evaluation of random forest regression for prediction of breeding value from genomewide SNPs. J. Genet..

[24] Farooq M, van Dijk ADJ, Nijveen H, Mansoor S, de Ridder D (2022). Genomic prediction in plants: Opportunities for ensemble machine learning based approaches. F1000Research.

[25] Barbosa IP (2021). Genome-enabled prediction through machine learning methods considering different levels of trait complexity. Crop Sci..

[26] da Costa WG (2022). Genomic prediction through machine learning and neural networks for traits with epistasis. Comput. Struct. Biotechnol. J..

[27] de Sousa IC (2021). Genomic prediction of leaf rust resistance to Arabica coffee using machine learning algorithms. Sci. Agric..

[28] Westhues CC (2021). Prediction of maize phenotypic traits with genomic and environmental predictors using gradient boosting frameworks. Front. Plant Sci..

[29] Silva KJ (2020). High-density SNP-based genetic diversity and heterotic patterns of tropical maize breeding lines. Crop Sci..

[30] Dias KODG (2018). Estimating genotype × environment interaction for and genetic correlations among drought tolerance traits in maize via factor analytic multiplicative mixed models. Crop Sci..

[31] Technow F (2014). Genome properties and prospects of genomic prediction of hybrid performance in a breeding program of maize. Genetics.

[32] Vitezica ZG, Varona L, Legarra A (2013). On the additive and dominant variance and covariance of individuals within the genomic selection scope. Genetics.

[33] Amadeu RR (2016). AGHmatrix: R package to construct relationship matrices for autotetraploid and diploid species: A blueberry example. Plant Genome.

[34] VanRaden PM (2008). Efficient methods to compute genomic predictions. J. Dairy Sci..

[35] Falconer, D. S. & Mackay, T. F. C. Introduction to quantitative genetics. Essex. *UK Longman Gr.* (1996).

[36] Gilmour, A. R., Gogel, B. J., Cullis, B. R., Welham, S. J. & Thompson, R. ASReml User Guide Release 4.2 Functional Specification. *VSN Int. Ltd* (2021).

[37] Corbeil RR, Searle SR (1976). Restricted maximum likelihood (REML) estimation of variance components in the mixed model. Technometrics.

[38] Wilks SS (1938). The large-sample distribution of the likelihood ratio for testing composite hypotheses. Ann. Math. Stat..

[39] Dobson A, Barnett A (2008). An Introduction to Generalized Linear Models.

[40] Ho, T. K. Random decision forests. In *Proceedings of 3rd International Conference on Document Analysis and Recognition* vol. 1 278–282 (IEEE, 1995).

[41] Breiman L (2001). Random forests. Mach. Learn..

[42] Greenwell, B., Boehmke, B., Cunningham, J. & GBM, D. gbm: Generalized boosted regression models. R package version 2.1. 5. *Website https//cran. r-project. org/package= gbm [accessed 12 January 2020]* (2019).

[43] R Core Team. R: A language and environment for statistical computing. at (2021).

[44] Resende, M. D. V. de, Silva, F. F. e & Azevedo, C. F. Estatística matemática, biométrica e computacional: Modelos mistos, multivariados, categóricos e generalizados (REML/BLUP), inferência bayesiana, regressão aleatória, seleção genômica, QTL-GWAS, estatística espacial e temporal, competição, sobrevivência. *Viçosa Ed. UFV* 1–881 (2014).

[45] Gezan SA, de Carvalho MP, Sherrill J (2017). Statistical methods to explore genotype-by-environment interaction for loblolly pine clonal trials. Tree Genet. Genomes.

[46] Fernandes SB, Dias KOG, Ferreira DF, Brown PJ (2018). Efficiency of multi-trait, indirect, and trait-assisted genomic selection for improvement of biomass sorghum. Theor. Appl. Genet..

[47] Nishio M, Satoh M (2014). Including dominance effects in the genomic BLUP method for genomic evaluation. PLoS One.

[48] Reif JC, Gumpert F-M, Fischer S, Melchinger AE (2007). Impact of interpopulation divergence on additive and dominance variance in hybrid populations. Genetics.

[49] Sprague, G. F. & Tatum, L. A. General vs. specific combining ability in single crosses of corn. *J. Am. Soc. Agron.* (1942).

[50] Giraud H (2017). Reciprocal genetics: Identifying QTL for general and specific combining abilities in hybrids between multiparental populations from two maize (*Zea mays* L.) heterotic groups. Genetics.

[51] Hofmarcher P, Grün B (2020). Macroeconomic Forecasting in the Era of Big Data.

[52] Myles AJ, Feudale RN, Liu Y, Woody NA, Brown SD (2004). An introduction to decision tree modeling. J. Chemom..

[53] Friedman JH (2001). Greedy function approximation: A gradient boosting machine. Ann. Stat..

[54] Westhues CC, Simianer H, Beissinger TM (2022). learnMET: an R package to apply machine learning methods for genomic prediction using multi-environment trial data. G3 Fenes Genomes Genetics.

[55] Ghafouri-Kesbi F, Rahimi-Mianji G, Honarvar M, Nejati-Javaremi A (2017). Predictive ability of random forests, boosting, support vector machines and genomic best linear unbiased prediction in different scenarios of genomic evaluation. Anim. Prod. Sci..

[56] Zhang X (2022). Genetic architecture of maize yield traits dissected by QTL mapping and GWAS in maize. Crop J..

[57] Zhang X (2020). A combination of linkage mapping and GWAS brings new elements on the genetic basis of yield-related traits in maize across multiple environments. Theor. Appl. Genet..

[58] Steinhoff J (2012). Detection of QTL for flowering time in multiple families of elite maize. Theor. Appl. Genet..

[59] Buckler ES (2009). The genetic architecture of maize flowering time. Science.

[60] Abdollahi-Arpanahi R, Gianola D, Peñagaricano F (2020). Deep learning versus parametric and ensemble methods for genomic prediction of complex phenotypes. Genet. Sel. Evol..

[61] Technow F, Riedelsheimer C, Schrag TA, Melchinger AE (2012). Genomic prediction of hybrid performance in maize with models incorporating dominance and population specific marker effects. Theor. Appl. Genet..

[62] Windhausen VS (2012). Effectiveness of genomic prediction of maize hybrid performance in different breeding populations and environments. G3 Genes Genomes Genet..

[63] Krchov L-M, Bernardo R (2015). Relative efficiency of genomewide selection for testcross performance of doubled haploid lines in a maize breeding program. Crop Sci..

[64] Massman JM, Gordillo A, Lorenzana RE, Bernardo R (2013). Genomewide predictions from maize single-cross data. Theor. Appl. Genet..

[65] Tech Services. Pricing brochure TSI 2023 test sites. *Bluffton IN:TechServices* https://techservicespro.com/test-locations/ (2023).

[66] University of Minnesota. Genotyping-by-sequencing (Pricing). *Genomics Center* https://genomics.umn.edu/service/standard-genotyping-sequencing (2023).

