# Supplementary material for: Genomic prediction in multi-environment trials in maize using statistical and machine learning methods
Source: Sci Rep. 2024 Jan 11;14:1062. doi: 10.1038/s41598-024-51792-3 (PMC10784464; doi:10.1038/s41598-024-51792-3)
Supplement: Supplementary file 1 — Supplementary Tables. [file 41598_2024_51792_MOESM1_ESM.docx]

**Table S1.** Predictive abilities analyzed within each environment and their respective standard errors for grain yield (GY) and female flowering time (FFT), for the four methodologies using CV1 scenario (tested hybrids were not evaluated in any environment) for the irrigated (WW) and water stress (WS) conditions.

| **CV1** | | | | | | | | | | | | |
| --- | --- | --- | --- | --- | --- | --- | --- | --- | --- | --- | --- | --- |
|  |  | **WS** | | | | |  | **WW** | | | | |
| Trait | Methodology | 2010 | |  | 2011 | |  | 2010 | |  | 2011 | |
|  |  | Janaúba | Teresina |  | Janaúba | Teresina |  | Janaúba | Teresina |  | Janaúba | Teresina |
| GY | *bagging* | 0.377±0.013 | 0.176±0.022 |  | 0.270±0.019 | 0.359±0.026 |  | 0.323±0.014 | 0.479±0.014 |  | 0.356±0.010 | 0.253±0.016 |
|  | *random* *forest* | 0.364±0.017 | 0.177±0.022 |  | 0.242±0.017 | 0.365±0.023 |  | 0.297±0.011 | 0.474±0.010 |  | 0.347±0.011 | 0.265±0.008 |
|  | *boosting* | 0.248±0.028 | 0.165±0.037 |  | 0.204±0.026 | 0.294±0.034 |  | 0.256±0.037 | 0.379±0.020 |  | 0.247±0.022 | 0.237±0.018 |
|  | *GBLUP-A* | 0.356±0.009 | 0.156±0.030 |  | 0.008±0.015 | 0.017±0.023 |  | 0.172±0.011 | 0.400±0.011 |  | 0.290-0.007 | 0.267±0.008 |
|  | *GBLUP-AD* | 0.464±0.010 | 0.194±0.015 |  | 0.454±0.015 | 0.438±0.021 |  | 0.421±0.013 | 0.507±0.008 |  | 0.367±0.011 | 0.309±0.010 |
| FFT | *bagging* | 0.593±0.021 | 0.158±0.018 |  | 0.545±0.014 | 0.296±0.004 |  | 0.555±0.027 | 0.647±0.005 |  | 0.352±0.023 | 0.416±0.020 |
|  | *random* *forest* | 0.610±0.027 | 0.173±0.018 |  | 0.564±0.013 | 0.345±0.008 |  | 0.565±0.031 | 0.662±0.006 |  | 0.380±0.019 | 0.458±0.018 |
|  | *boosting* | 0.529±0.030 | 0.112±0.029 |  | 0.573±0.009 | 0.238±0.023 |  | 0.497±0.028 | 0.640±0.006 |  | 0.366±0.027 | 0.462±0.023 |
|  | *GBLUP-A* | 0.619±0.026 | 0.218±0.010 |  | 0.580±0.018 | 0.267±0.023 |  | 0.536±0.021 | 0.666±0.006 |  | 0.436±0.020 | 0.450±0.023 |
|  | *GBLUP-AD* | 0.648±0.023 | 0.202±0.017 |  | 0.633±0.019 | 0.410±0.014 |  | 0.580±0.028 | 0.689±0.007 |  | 0.419±0.019 | 0.534±0.021 |

**Table S2.** Predictive abilities analyzed within each environment and their respective standard errors for grain yield (GY) and female flowering time (FFT), for the four methodologies using CV2-50% scenario (tested hybrids were not evaluated at 50% environments), for irrigated (WW) and water stress (WS) conditions.

| **CV2 (50%)** | | | | | | | | | | | | |
| --- | --- | --- | --- | --- | --- | --- | --- | --- | --- | --- | --- | --- |
|  |  | **WS** | | | | |  | **WW** | | | | |
| Trait | Methodology | 2010 | |  | 2011 | |  | 2010 | |  | 2011 | |
|  |  | Janaúba | Teresina |  | Janaúba | Teresina |  | Janaúba | Teresina |  | Janaúba | Teresina |
| GY | *bagging* | 0.449±0.007 | 0.345±0.025 |  | 0.358±0.008 | 0.505±0.015 |  | 0.347±0.021 | 0.426±0.015 |  | 0.344±0.023 | 0.243±0.006 |
|  | *random* *forest* | 0.412±0.010 | 0.351±0.027 |  | 0.355±0.017 | 0.493±0.020 |  | 0.336±0.021 | 0.412±0.011 |  | 0.337±0.023 | 0.243±0.004 |
|  | *boosting* | 0.334±0.015 | 0.306±0.023 |  | 0.337±0.035 | 0.436±0.024 |  | 0.334±0.021 | 0.422±0.015 |  | 0.347±0.020 | 0.198±0.012 |
|  | *GBLUP-A* | 0.429±0.007 | 0.285±0.022 |  | 0.172±0.023 | 0.226±0.018 |  | 0.269±0.016 | 0.455±0.002 |  | 0.337±0.005 | 0.284±0.007 |
|  | *GBLUP-AD* | 0.517±0.007 | 0.295±0.020 |  | 0.506±0.020 | 0.534±0.017 |  | 0.449±0.022 | 0.544±0.003 |  | 0.414±0.011 | 0.305±0.010 |
| FFT | *bagging* | 0.595±0.020 | 0.178±0.005 |  | 0.549±0.014 | 0.259±0.028 |  | 0.556±0.026 | 0.671±0.007 |  | 0.358±0.021 | 0.496±0.031 |
|  | *random* *forest* | 0.624±0.032 | 0.187±0.015 |  | 0.609±0.012 | 0.337±0.034 |  | 0.607±0.032 | 0.700±0.009 |  | 0.491±0.012 | 0.564±0.012 |
|  | *boosting* | 0.557±0.028 | 0.210±0.015 |  | 0.612±0.024 | 0.364±0.032 |  | 0.592±0.015 | 0.687±0.008 |  | 0.537±0.009 | 0.573±0.008 |
|  | *GBLUP-A* | 0.692±0.017 | 0.257±0.004 |  | 0.632±0.013 | 0.414±0.018 |  | 0.646±0.007 | 0.719±0.003 |  | 0.553±0.008 | 0.570±0.006 |
|  | *GBLUP-AD* | 0.709±0.016 | 0.243±0.007 |  | 0.673±0.013 | 0.493±0.025 |  | 0.670±0.010 | 0.734±0.003 |  | 0.548±0.008 | 0.612±0.008 |

**Table S3.** Predictive abilities analyzed within each environment and their respective standard errors for grain yield (GY) and female flowering time (FFT), for the four methodologies using CV2-25% scenario (tested hybrids were not evaluated at 25% environments), for irrigated (WW) and water stress (WS) conditions.

| **CV2 (25%)** | | | | | | | | | | | | |
| --- | --- | --- | --- | --- | --- | --- | --- | --- | --- | --- | --- | --- |
|  |  | **WS** | | | | |  | **WW** | | | | |
| Trait | Methodology | 2010 | |  | 2011 | |  | 2010 | |  | 2011 | |
|  |  | Janaúba | Teresina |  | Janaúba | Teresina |  | Janaúba | Teresina |  | Janaúba | Teresina |
| GY | *bagging* | 0.462±0.003 | 0.385±0.004 |  | 0.393±0.006 | 0.539±0.002 |  | 0.382±0.002 | 0.455±0.003 |  | 0.326±0.005 | 0.245±0.006 |
|  | *random* *forest* | 0.429±0.003 | 0.399±0.002 |  | 0.395±0.003 | 0.536±0.002 |  | 0.377±0.001 | 0.447±0.002 |  | 0.332±0.003 | 0.248±0.003 |
|  | *boosting* | 0.361±0.003 | 0.341±0.003 |  | 0.376±0.006 | 0.481±0.004 |  | 0.365±0.008 | 0.466±0.006 |  | 0.343±0.006 | 0.223±0.008 |
|  | *GBLUP-A* | 0.452±0.005 | 0.326±0.012 |  | 0.245±0.016 | 0.281±0.018 |  | 0.292±0.013 | 0.469±0.004 |  | 0.346±0.005 | 0.288±0.007 |
|  | *GBLUP-AD* | 0.524±0.004 | 0.315±0.011 |  | 0.524±0.005 | 0.552±0.008 |  | 0.466±0.005 | 0.555±0.003 |  | 0.418±0.010 | 0.304±0.010 |
| FFT | *bagging* | 0.594±0.022 | 0.159±0.012 |  | 0.550±0.014 | 0.210±0.005 |  | 0.558±0.027 | 0.684±0.004 |  | 0.347±0.021 | 0.532±0.009 |
|  | *random* *forest* | 0.667±0.015 | 0.177±0.009 |  | 0.641±0.004 | 0.304±0.005 |  | 0.654±0.012 | 0.707±0.002 |  | 0.517±0.008 | 0.591±0.004 |
|  | *boosting* | 0.586±0.006 | 0.205±0.006 |  | 0.662±0.008 | 0.363±0.006 |  | 0.614±0.005 | 0.694±0.006 |  | 0.573±0.004 | 0.590±0.004 |
|  | *GBLUP-A* | 0.709±0.007 | 0.263±0.004 |  | 0.657±0.008 | 0.432±0.007 |  | 0.669±0.004 | 0.730±0.003 |  | 0.576±0.005 | 0.591±0.002 |
|  | *GBLUP-AD* | 0.719±0.005 | 0.247±0.009 |  | 0.696±0.006 | 0.502±0.006 |  | 0.688±0.008 | 0.745±0.003 |  | 0.568±0.007 | 0.629±0.003 |

**Table S4.** Mean predictive abilities and their respective standard errors for grain yield (GY) and female flowering time (FFT), for the four methodologies, using CV1, CV2 (50%) and CV2 (25%) scenarios, for the irrigated (WW) and water stress (WS) conditions.

| Trait | Methodology |  | **WS** | |  |  | **WW** | |  |
| --- | --- | --- | --- | --- | --- | --- | --- | --- | --- |
|  |  |  | CV1 | CV2 (50%) | CV2 (25%) |  | CV1 | CV2 (50%) | CV2 (25%) |
| GY | *bagging* |  | 0.296±0.046 | 0.414±0.038 | 0.445±0.036 |  | 0.311±0.026 | 0.340±0.037 | 0.352±0.044 |
|  | *random* *forest* |  | 0.287±0.047 | 0.403±0.033 | 0.440±0.033 |  | 0.346±0.046 | 0.332±0.035 | 0.351±0.042 |
|  | *boosting* |  | 0.228±0.028 | 0.353±0.028 | 0.390±0.031 |  | 0.280±0.033 | 0.325±0.047 | 0.349±0.050 |
|  | *GBLUP-A* |  | 0.134±0.081 | 0.278±0.055 | 0.326±0.045 |  | 0.282±0.047 | 0.336±0.042 | 0.349±0.042 |
|  | *GBLUP-AD* |  | 0.388±0.065 | 0.463±0.056 | 0.479±0.055 |  | 0.401±0.042 | 0.428±0.049 | 0.436±0.052 |
| FFT | *bagging* |  | 0.398±0.103 | 0.395±0.104 | 0.378±0.113 |  | 0.493±0.067 | 0.520±0.065 | 0.530±0.070 |
|  | *random* *forest* |  | 0.423±0.101 | 0.439±0.107 | 0.447±0.122 |  | 0.516±0.062 | 0.591±0.044 | 0.617±0.041 |
|  | *boosting* |  | 0.363±0.112 | 0.436±0.092 | 0.454±0.104 |  | 0.491±0.057 | 0.597±0.032 | 0.618±0.027 |
|  | *GBLUP-A* |  | 0.421±0.104 | 0.499±0.100 | 0.515±0.103 |  | 0.522±0.053 | 0.622±0.038 | 0.642±0.036 |
|  | *GBLUP-AD* |  | 0.4730.106 | 0.530±0.107 | 0.541±0.109 |  | 0.556±0.056 | 0.641±0.040 | 0.658±0.038 |
